# Development and validation of a prognostic model for acute respiratory distress syndrome in critically Ill patients with intra-abdominal sepsis: a multicenter cohort study

**DOI:** 10.3389/fmed.2026.1775636

**Published:** 2026-03-12

**Authors:** Wenzhe Li, Yuqian Li, Yixi Wang, Jian Cui, Xiangyou Yu

**Affiliations:** 1 Department of Critical Care Medicine, The First Affiliated Hospital of Xinjiang Medical University, Urumqi, China; 2 Department of Anesthesiology, The First Affiliated Hospital of Xinjiang Medical University, Urumqi, China; 3 Department of Minimally Invasive Spine and Precision Orthopedics, The First Affiliated Hospital of Xinjiang Medical University, Urumqi, China

**Keywords:** acute respiratory distress syndrome (ARDS), intra-abdominal sepsis, mechanical ventilation, Sequential Organ Failure Assessment (SOFA) score, stacking model

## Abstract

**Background:**

To develop and externally validate a machine learning-based model for predicting the risk of acute respiratory distress syndrome (ARDS) in patients with intra-abdominal sepsis.

**Methods:**

Data were obtained from the MIMIC-IV and the eICU-CRD database, including patients diagnosed with intra-abdominal sepsis. ARDS occurrence during intensive care unit (ICU) stay was defined as the primary outcome. Feature selection was performed using a combination of the Boruta algorithm, LASSO regression, and logistic regression. Ten base machine learning algorithms were trained and integrated into a stacked ensemble model. Model performance was systematically evaluated, and interpretability was assessed using SHapley Additive exPlanations (SHAP). External validation was conducted in an independent cohort of patients with intra-abdominal sepsis admitted to the First Affiliated Hospital of Xinjiang Medical University between 2016 and 2024. A web-based risk prediction calculator was subsequently developed to facilitate clinical decision support.

**Results:**

Among 1,120 patients included from the MIMIC-IV and eICU-CRD databases, 554 (49.46%) developed ARDS during their ICU stay. Fourteen predictors were retained, including mechanical ventilation, use of vasoactive agents, history of chronic pulmonary disease, Sequential Organ Failure Assessment (SOFA) score, Glasgow Coma Scale (GCS) score, key vital signs, and routine laboratory indicators. The stacking model achieved areas under the receiver operating characteristic curve (AUC) of 0.811 in the development cohort, 0.794 in the internal validation cohort, and 0.756 in the external validation cohort. SHAP analysis identified mechanical ventilation as the most influential predictor, while early vasoactive agents use was associated with a reduced ARDS risk.

**Conclusion:**

A stacked ensemble model for predicting ARDS risk in patients with intra-abdominal sepsis demonstrated robust performance, stability, and interpretability. This model provides a practical tool for early risk stratification and informed clinical decision-making.

## 1 Introduction

Sepsis is a life-threatening syndrome characterized by acute organ dysfunction resulting from a dysregulated host immune response to infection (1). Among its etiologies, intra-abdominal infection represents a common and severe clinical condition that frequently progresses to systemic infection. It is the second most prevalent cause of sepsis after pulmonary infection and constitutes a substantial global health burden (2, 3). Disruption of the intra-abdominal anatomical barrier facilitates the translocation of pathogens into the bloodstream and lymphatic system, thereby triggering an exaggerated host inflammatory response (4). The excessive release of inflammatory mediators can lead to vascular endothelial injury, increased capillary permeability, and impaired tissue perfusion, ultimately culminating in multiple organ dysfunction (5). Acute respiratory distress syndrome (ARDS) secondary to sepsis is a particularly severe complication with a highly complex and multifactorial pathogenesis. Despite its profound impact on morbidity and mortality, effective disease-specific therapeutic interventions remain limited (6, 7). Consequently, early identification of patients at high risk for ARDS, coupled with individualized treatment strategies, has the potential to substantially improve clinical outcomes. Previous studies have reported that factors such as advanced age, elevated procalcitonin level, and biliary tract infections are associated with an increased risk of ARDS in patients with intra-abdominal sepsis (8). However, the clinical specificity and predictive utility of these indicators remain suboptimal. In recent years, the application of machine learning techniques in disease risk prediction has gained increasing attention. In particular, classification models capable of distinguishing inflammatory subphenotypes in ARDS have enabled more precise patient stratification and informed targeted therapeutic strategies (9). Against this backdrop, the present study sought to develop an early prediction model for ARDS in patients with intra-abdominal sepsis using machine learning algorithms applied to routinely collected clinical data. By enabling timely identification of high-risk individuals, this approach aims to support early diagnostic and therapeutic decision-making and ultimately improve patient prognosis.

## 2 Materials and methods

### 2.1 Sources of data

Data for this study were derived from the Medical Information Mart for Intensive Care IV (MIMIC-IV, version 3.1) and the eICU Collaborative Research Database (eICU-CRD, version 2.0) to develop a risk prediction model for ARDS in patients with intra-abdominal sepsis. The MIMIC-IV database is a large, publicly accessible critical care dataset developed and maintained by the Massachusetts Institute of Technology and is widely used in critical illness research (10). The eICU-CRD is a multicenter database that includes detailed clinical information from more than 200,000 critically ill patients admitted to 208 hospitals across the United States between 2014 and 2015 (11). Access to both databases was granted following completion of the required data use training and authorization procedures (certification number: 57264471), and relevant data were subsequently extracted for analysis. To further evaluate the generalizability of the prediction model, an external validation cohort comprising patients with intra-abdominal sepsis admitted to the intensive care unit of the First Affiliated Hospital of Xinjiang Medical University (XJMU) was included. The study protocol was reviewed and approved by the Ethics Committee of the First Affiliated Hospital of Xinjiang Medical University (approval number: K202404-45).

### 2.2 Population selection criteria

**FIGURE 1:**
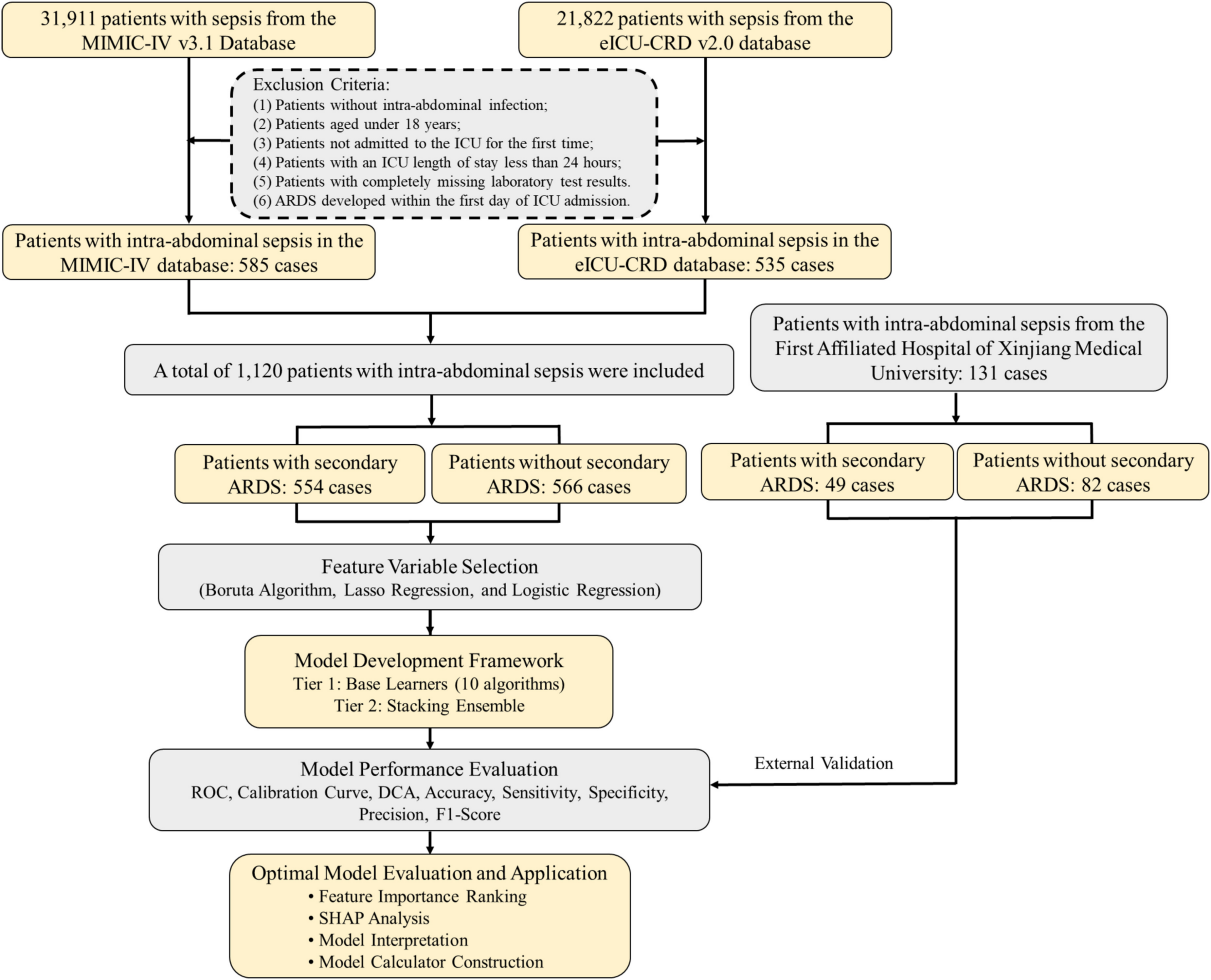
Screening and inclusion process of intra-abdominal sepsis patients for analysis.

Patients were screened from the MIMIC-IV and eICU-CRD databases in accordance with the Sepsis-3 criteria, which define sepsis as a confirmed or suspected infection accompanied by a Sequential Organ Failure Assessment (SOFA) score of ≥2, or an increase of at least two points from baseline (1). Eligible participants were adults with a definitive diagnosis of intra-abdominal infection complicated by sepsis. Given that both the MIMIC-IV and eICU-CRD databases utilize ICD-9 and ICD-10 coding systems (12), we included patients with either the original ICD-9 codes from prior studies or their clinically equivalent ICD-10 codes, ensuring consistency in infection identification across all systems. A full list of the relevant ICD-9 and ICD-10 codes for intra-abdominal infections is provided in Supplementary Table 1. In addition, an independent cohort of patients with the same diagnosis admitted between 2016 and 2024 was identified from the electronic medical record (EMR) system of the First Affiliated Hospital of XJMU for external validation. Patients were excluded if they met any of the following criteria: (1) age < 18 years; (2) admission to the intensive care unit (ICU) was not the first during the index hospitalization; (3) ICU length of stay < 24 h; or (4) absence of laboratory test results for all measured variables; (4) ARDS developed within the first 24 h of ICU admission. A detailed flowchart of patient selection is presented in Figure 1.

### 2.3 Data screening and feature extraction

Patient data were extracted using Structured Query Language (SQL) via PostgreSQL 16^1^ and Navicat Premium 17^2^. The dataset comprised routinely collected vital signs, including heart rate, blood pressure, respiratory rate, pulse oxygen saturation (SpO_2_), and body temperature, as well as laboratory measurements obtained within the first 24 h following ICU admission. A comprehensive set of candidate variables was identified for analysis, encompassing baseline demographic characteristics (e.g., age, sex, height, weight, and race) and established disease severity and comorbidity scores, including the Charlson Comorbidity Index, SOFA score, Acute Physiology and Chronic Health Evaluation II (APACHE II) score, and Glasgow Coma Scale (GCS) score. ARDS was defined according to the Berlin Definition using standardized operational criteria for timing, oxygenation (PaO_2_/FiO_2_ ≤ 300 mmHg with PEEP ≥ 5 cmH_2_O), radiographic findings, and exclusion of hydrostatic pulmonary edema (13). The detailed variable mapping across cohorts is provided in Supplementary Table 2. The primary outcome of this study was the development of ARDS occurring after the first 24 h of ICU admission. All laboratory values and severity scores were derived from data recorded during the first 24 h of ICU admission to ensure consistency in early risk assessment.

### 2.4 Statistical analysis and model construction

Quantitative variables were first assessed for normality. Normally distributed data are presented as mean ± standard deviation and were compared using the independent-samples *t*-test, whereas non-normally distributed variables are reported as median [min, max] and were analyzed using the Wilcoxon rank-sum test. Categorical variables are expressed as frequencies and percentages and were compared using the chi-square test. Patients were stratified based on the occurrence of ARDS. The MIMIC-IV and eICU-CRD datasets were split into a training set (70%) and an internal validation set (30%). Variables with > 20% missing data were excluded, while missing values in variables with ≤ 20% missingness were imputed using multiple imputation by chained equations (MICE), performed exclusively within the training set.

**FIGURE 2:**
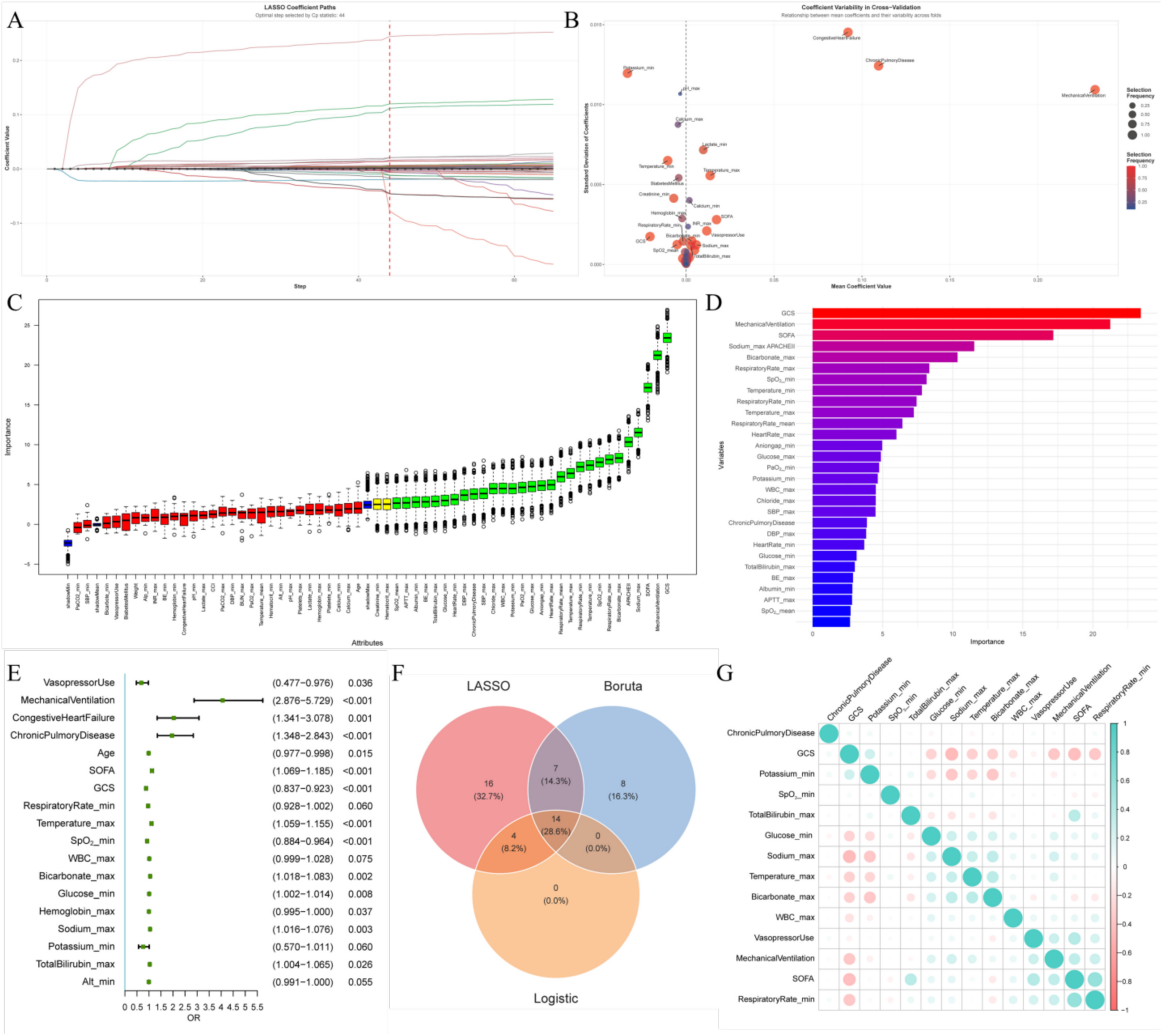
Feature selection using least absolute shrinkage and selection operator (LASSO) regression, the Boruta algorithm, and logistic regression. **(A)** Feature selection using LASSO regression; **(B)** relative importance of the 41 features identified by LASSO regression; **(C)** feature evaluation using the Boruta algorithm; **(D)** importance ranking of the 29 features selected by the Boruta algorithm; **(E)** importance of the 18 features identified by logistic regression; **(F)** cross-validation results identifying the 14 most informative features retained for model construction; **(G)** multicollinearity assessment of the 14 selected features.

Feature selection was performed by identifying the intersection of variables selected using three complementary approaches: the Boruta algorithm, least absolute shrinkage and selection operator (LASSO) regression, and logistic regression (Figure 2). Based on the selected features, 10 machine learning models were trained, including Adaptive Boosting (AdaBoost), Categorical Boosting (CatBoost), Decision Tree (DT), Gradient Boosting Machine (GBM), k-Nearest Neighbors (KNN), Logistic Regression (LR), Multilayer Perceptron (MLP), Random Forest (RF), Support Vector Machine (SVM), and Extreme Gradient Boosting (XGBoost), the model parameter settings were summarized in Supplementary Table 3. A stacked ensemble model was constructed using a stacked generalization (Stacking) framework. The predictions from multiple base models were used as features for a Logistic Regression meta-model, which combined them to make the final prediction. Model performance was evaluated using receiver operating characteristic (ROC) curves with calculation of the area under the curve (AUC), along with decision curve analysis (DCA), calibration curves, and precision–recall (PR) curves. Additional performance metrics included accuracy, precision, sensitivity, specificity, and the F1 score. The stacking model was selected for Shapley Additive Explanations (SHAP) analysis. The generalizability of the model was assessed using an external validation dataset. To facilitate clinical implementation, an online prediction tool was developed.

All statistical analyses were performed using R software (version 4.4.3) and Python (version 3.10). A two-sided *p* < 0.05 was considered statistically significant.

## 3 Results

### 3.1 Baseline characteristics

A total of 1,120 patients with intra-abdominal sepsis were included in the analysis, among whom 554 (49.46%) developed ARDS during their ICU stay. Compared with patients who did not develop ARDS, those with ARDS exhibited significantly higher APACHE II and SOFA score, as well as a greater Charlson Comorbidity Index. In addition, ARDS patients demonstrated lower GCS score and a higher prevalence of comorbid conditions, including congestive heart failure and chronic pulmonary disease. Notable hemodynamic differences were observed between the two groups. Patients with ARDS presented with lower systolic and diastolic blood pressure, accompanied by higher heart rate and respiratory rate values (all *p* < 0.05) (Table 1). Significant between-group differences were also identified in multiple laboratory parameters, including white blood cell count, hematocrit, hemoglobin, blood urea nitrogen, serum creatinine, serum calcium, peak blood glucose levels, and arterial blood gas measurements (all *p* < 0.05) (Table 2). Furthermore, patients who developed ARDS had a higher frequency of mechanical ventilation and use of vasoactive agents. These patients experienced substantially worse clinical outcomes, including higher 28-day, in-hospital, and ICU mortality rates, as well as significantly prolonged ICU and total hospital lengths of stay compared with non-ARDS patients (all *p* < 0.05) (Table 1).

**TABLE 1:** Characteristics of the study population in the Medical Information Mart for Intensive Care IV (MIMIC-IV) and eICU Collaborative Research Database (eICU-CRD) cohorts.

Variables	All patients (*n* = 1120)	Non-ARDS patients (*n* = 556)	ARDS patients (*n* = 554)	*P*-value
Age (years)	62.4 ± 15.0	63.7 ± 15.3	61.1 ± 14.6	0.004
Gender (male)	643 (57.4%)	318 (56.2%)	325 (58.7%)	0.436
Height (cm)	170 ± 10.9	170 ± 10.9	170 ± 10.9	0.952
Weight (kg)	84.2 ± 25.3	81.8 ± 23.6	86.6 ± 26.7	0.001
**Race**				
White	833 (74.4%)	409 (72.3%)	424 (76.5%)	0.145
Black	86 (7.7%)	48 (8.5%)	38 (6.9%)	–
Asian	89 (7.9%)	54 (9.5%)	35 (6.3%)	–
Other	112 (10.0%)	55 (9.7%)	57 (10.3%)	–
Charlson comorbidity index	5.2 ± 2.9	5.5 ± 3.0	5.0 ± 2.8	0.002
SOFA score	8.4 ± 4.3	6.9 ± 3.6	9.8 ± 4.4	<0.001
APACHE II score	22.1 ± 8.3	19.9 ± 7.4	24.3 ± 8.5	<0.001
GCS score	11.7 ± 4.1	13.4 ± 2.6	10.0 ± 4.6	<0.001
**Comorbidities**				
Congestive heart failure	173 (15.4%)	75 (13.3%)	98 (17.7%)	0.049
Peripheral vascular disease	76 (6.8%)	40 (7.1%)	36 (6.5%)	0.795
Cerebrovascular disease	51 (4.6%)	23 (4.1%)	28 (5.1%)	0.515
Chronic pulmonary disease	203 (18.1%)	81 (14.3%)	122 (22.0%)	0.001
Rheumatic disease	40 (3.6%)	23 (4.1%)	17 (3.1%)	0.462
Diabetes	298 (26.6%)	156 (27.6%)	142 (25.6%)	0.507
Chronic kidney disease	266 (23.8%)	141 (24.9%)	125 (22.6%)	0.394
Metastatic solid tumor	81 (7.2%)	49 (8.7%)	32 (5.8%)	0.081
**Vital signs**				
Minimum heart rate (beats/min)	72.9 ± 17.9	74.9 ± 17.2	70.8 ± 18.4	<0.001
Maximum heart rate (beats/min)	118 ± 24.1	113 ± 23.3	123 ± 24.0	<0.001
Minimum systolic blood pressure (mmHg)	81.6 ± 17.5	84.1 ± 17.0	79.2 ± 17.6	<0.001
Maximum systolic blood pressure (mmHg)	150 ± 27.9	145 ± 25.7	155 ± 29.1	<0.001
Minimum diastolic blood pressure (mmHg)	41.5 ± 11.8	43.3 ± 11.9	39.7 ± 11.4	<0.001
Maximum diastolic blood pressure (mmHg)	91.7 ± 24.3	88.5 ± 23.7	94.9 ± 24.6	<0.001
Minimum respiratory rate (breaths/min)	11.2 ± 5.2	12.1 ± 4.4	10.3 ± 5.8	<0.001
Maximum respiratory rate (breaths/min)	31.6 ± 8.5	29.5 ± 7.6	33.7 ± 8.8	<0.001
Minimum temperature (°C)	35.9 ± 1.1	36.1 ± 1.0	35.8 ± 1.2	<0.001
Maximum temperature (°C)	37.7 ± 0.9	37.5 ± 0.8	37.9 ± 1.0	<0.001
Minimum SpO_2_ (%)	86.2 ± 15.3	89.0 ± 11.8	83.2 ± 17.7	<0.001
Maximum SpO_2_ (%)	99.5 ± 1.43	99.5 ± 0.988	99.6 ± 1.76	0.24
Use of vasopressors	427 (38.1%)	187 (33.0%)	240 (43.3%)	<0.001
Use of mechanical ventilation	388 (34.6%)	93 (16.4%)	295 (53.2%)	<0.001
Total length of stay (days)	19.0 ± 20.6	17.2 ± 20.5	20.8 ± 20.5	0.004
ICU length of stay (days)	7.2 ± 10.0	5.0 ± 8.6	9.4 ± 10.8	<0.001
28-day mortality	315 (28.1%)	128 (22.6%)	187 (33.8%)	<0.001
In-hospital mortality	305 (27.2%)	111 (19.6%)	194 (35.0%)	<0.001
ICU mortality	196 (17.5%)	60 (10.6%)	136 (24.5%)	<0.001

Data shown as mean ± SD, or numbers with %.

**TABLE 2:** Laboratory parameters recorded within the first 24 h of ICU admission in the Medical Information Mart for Intensive Care IV (MIMIC-IV) and eICU Collaborative Research Database (eICU-CRD) cohorts.

Variables	All patients (*n* = 1120)	Non-ARDS patients (*n* = 556)	ARDS patients (*n* = 554)	*P*-value
Minimum white blood cell count (10^9^/L)	9.84 ± 7.38	9.89 ± 7.14	9.79 ± 7.61	0.821
Maximum white blood cell count (10^9^/L)	18.80 ± 10.90	17.20 ± 10.40	20.40 ± 11.20	<0.001
Minimum hemoglobin (g/dL)	8.63 ± 2.09	8.88 ± 2.11	8.38 ± 2.05	<0.001
Maximum hemoglobin (g/dL)	11.40 ± 2.37	11.20 ± 2.21	11.60 ± 2.51	0.008
Minimum hematocrit (%)	26.30 ± 6.19	27.00 ± 6.17	25.60 ± 6.13	<0.001
Maximum hematocrit (%)	34.50 ± 7.07	33.90 ± 6.56	35.10 ± 7.52	0.005
Minimum platelet count (10^9^/L)	148 ± 114	159 ± 119	137 ± 107	<0.001
Maximum platelet count (10^9^/L)	251 ± 173	235 ± 156	268 ± 188	0.001
Minimum blood urea nitrogen (mg/dL)	28.90 ± 24.50	30.30 ± 25.30	27.40 ± 23.60	0.053
Maximum blood urea nitrogen (mg/dL)	47.40 ± 32.70	45.2 ± 32.40	49.50 ± 32.90	0.026
Minimum serum creatinine (mg/dL)	1.66 ± 1.71	1.82 ± 1.90	1.49 ± 1.49	0.001
Maximum serum creatinine (mg/dL)	2.68 ± 2.60	2.74 ± 2.79	2.61 ± 2.38	0.386
Minimum serum albumin (g/dL)	2.50 ± 0.73	2.59 ± 0.69	2.40 ± 0.75	<0.001
Maximum serum albumin (g/dL)	2.96 ± 0.72	2.93 ± 0.71	3.00 ± 0.74	0.092
Minimum sodium (mmol/L)	133 ± 4.43	133 ± 4.46	134 ± 4.40	0.138
Maximum sodium (mmol/L)	140 ± 5.31	139 ± 4.57	142 ± 5.59	<0.001
Minimum chloride (mmol/L)	98.80 ± 7.46	98.80 ± 7.66	98.80 ± 7.26	0.929
Maximum chloride (mmol/L)	108 ± 8.28	106 ± 7.14	109 ± 9.05	<0.001
Minimum potassium (mmol/L)	3.62 ± 0.55	3.71 ± 0.56	3.52 ± 0.53	<0.001
Maximum potassium (mmol/L)	4.78 ± 0.69	4.75 ± 0.68	4.81 ± 0.69	0.127
Minimum calcium (mg/dL)	7.48 ± 1.15	7.61 ± 1.05	7.34 ± 1.23	<0.001
Maximum calcium (mg/dL)	8.78 ± 1.00	8.68 ± 0.96	8.88 ± 1.07	<0.001
Minimum pH	7.28 ± 0.10	7.30 ± 0.09	7.27 ± 0.10	<0.001
Maximum pH	7.41 ± 0.07	7.40 ± 0.07	7.41 ± 0.07	0.003
Minimum PaO_2_ (mmHg)	80.80 ± 28.40	85.60 ± 28.40	76.00 ± 27.70	<0.001
Maximum PaO_2_ (mmHg)	175 ± 95.80	170 ± 95.30	181 ± 95.90	0.044
Minimum PaCO2 (mmHg)	32.90 ± 6.04	33.30 ± 5.78	32.50 ± 6.27	0.017
Maximum PaCO2 (mmHg)	43.70 ± 9.27	42.70 ± 9.04	44.60 ± 9.40	<0.001
Minimum BE	−6.37 ± 5.45	−5.97 ± 5.15	−6.78 ± 5.72	0.013
Maximum BE	−1.39 ± 4.59	−1.86 ± 4.27	−0.92 ± 4.84	<0.001
Minimum HCO_3_^–^	18.10 ± 5.10	18.50 ± 4.85	17.80 ± 5.31	0.025
Maximum HCO_3_^–^	24.70 ± 5.77	23.60 ± 5.03	25.80 ± 6.25	<0.001
Minimum anion gap	10.50 ± 4.76	11.00 ± 4.51	9.94 ± 4.94	<0.001
Maximum anion gap	16.60 ± 4.99	16.60 ± 4.93	16.70 ± 5.06	0.853
Minimum lactate (mmol/L)	1.94 ± 1.18	1.86 ± 1.03	2.02 ± 1.32	0.026
Maximum lactate (mmol/L)	3.67 ± 2.53	3.40 ± 2.43	3.95 ± 2.61	<0.001
Minimum blood glucose (mg/dL)	92.30 ± 27.40	94.80 ± 26.80	89.70 ± 27.80	0.002
Maximum blood glucose (mg/dL)	194 ± 73.20	184 ± 74.00	204 ± 71.20	<0.001
Minimum alanine aminotransferase (U/L)	33.50 ± 35.00	35.50 ± 37.40	31.40 ± 32.40	0.049
Maximum alanine aminotransferase (U/L)	79.20 ± 126.00	76.70 ± 133.00	81.80 ± 118.00	0.499
Minimum aspartate aminotransferase (U/L)	55.00 ± 61.40	57.40 ± 62.70	52.40 ± 60.10	0.176
Maximum aspartate aminotransferase (U/L)	149 ± 262	142 ± 262	157 ± 263	0.346
Minimum alkaline phosphatase (U/L)	94.50 ± 57.70	99.00 ± 58.70	89.90 ± 56.30	0.008
Maximum alkaline phosphatase (U/L)	138 ± 87.80	133 ± 85.10	142 ± 90.30	0.081
Minimum total bilirubin (mg/dl)	3.14 ± 4.96	3.05 ± 4.57	3.22 ± 5.34	0.575
Maximum total bilirubin (mg/dl)	4.40 ± 5.76	4.02 ± 5.20	4.80 ± 6.26	0.024
Maximum INR	2.00 ± 0.88	1.94 ± 0.84	2.06 ± 0.92	0.019
Maximum APTT (s)	46.00 ± 19.70	44.80 ± 19.20	47.20 ± 20.00	0.049

Data shown as mean ± SD.

### 3.2 Feature variable selection

To identify clinically relevant predictors associated with increased ARDS risk, candidate variables with a *P*-value < 0.05 were subjected to feature selection using three complementary algorithms. LASSO regression identified 41 candidate features (Figures 2A,B), the Boruta algorithm retained 29 features (Figures 2C,D), and logistic regression screening yielded 18 features (Figure 2E). Following cross-validation and integration of results across the three methods, 14 variables were selected as the most informative predictors: mechanical ventilation, use of vasoactive agents, history of chronic lung disease, SOFA score, GCS score, minimum respiratory rate, minimum oxygen saturation, maximum body temperature, maximum white blood cell count, maximum total bilirubin, maximum bicarbonate, minimum blood glucose, maximum serum sodium, and minimum serum potassium (Figure 2F). Multicollinearity assessment revealed no significant collinearity among the selected predictors, supporting the inclusion of all 14 variables in the final model construction (Figure 2G).

### 3.3 Model construction

**FIGURE 3:**
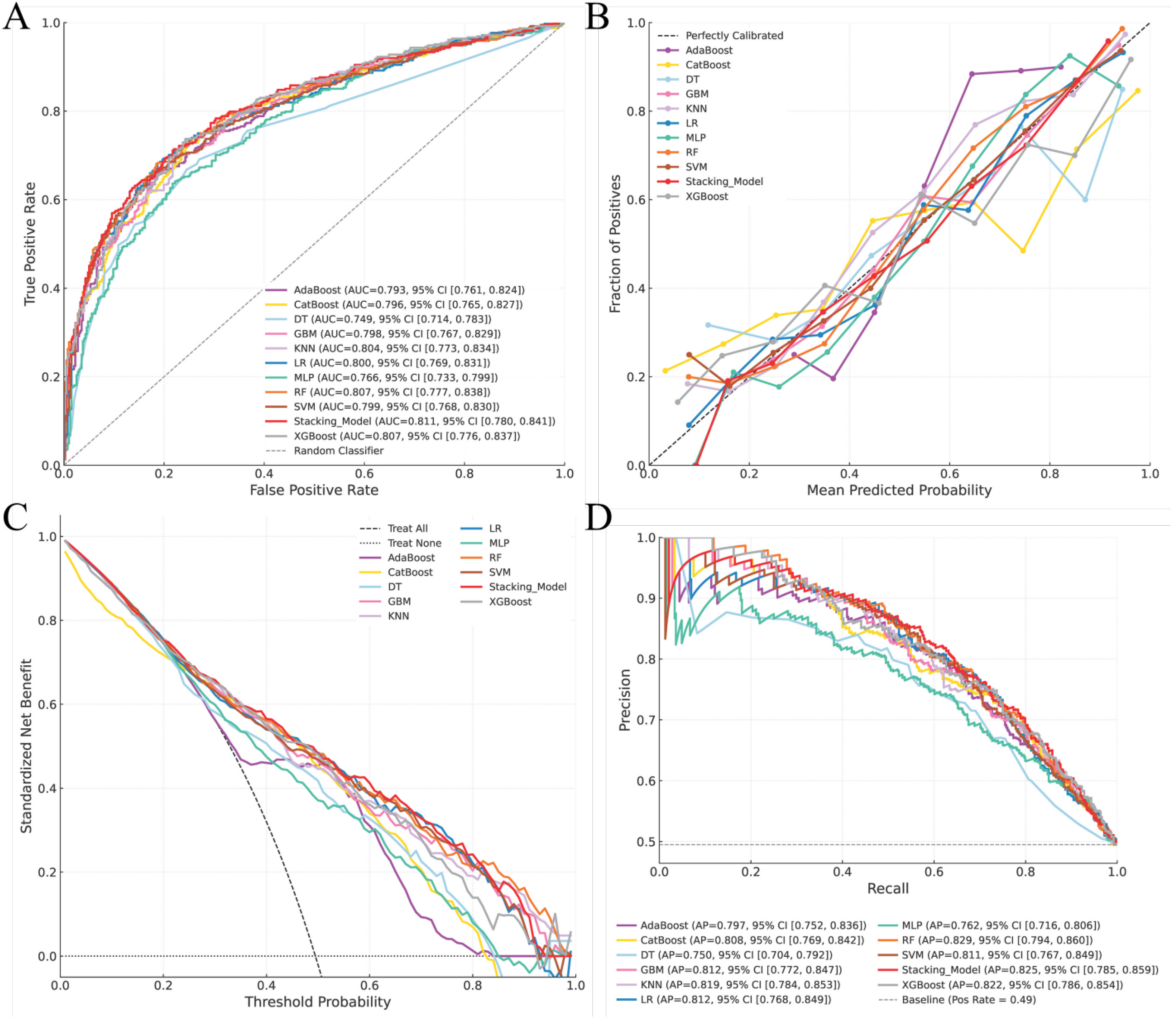
Construction and performance of the prediction model in the training cohort. **(A)** Receiver operating characteristic (ROC) curve for the training cohort; **(B)** calibration curve assessing agreement between predicted and observed outcomes in the training cohort; **(C)** decision curve analysis (DCA) evaluating the net clinical benefit across threshold probabilities in the training cohort; **(D)** precision–recall (PR) curve for the training cohort.

**FIGURE 4:**
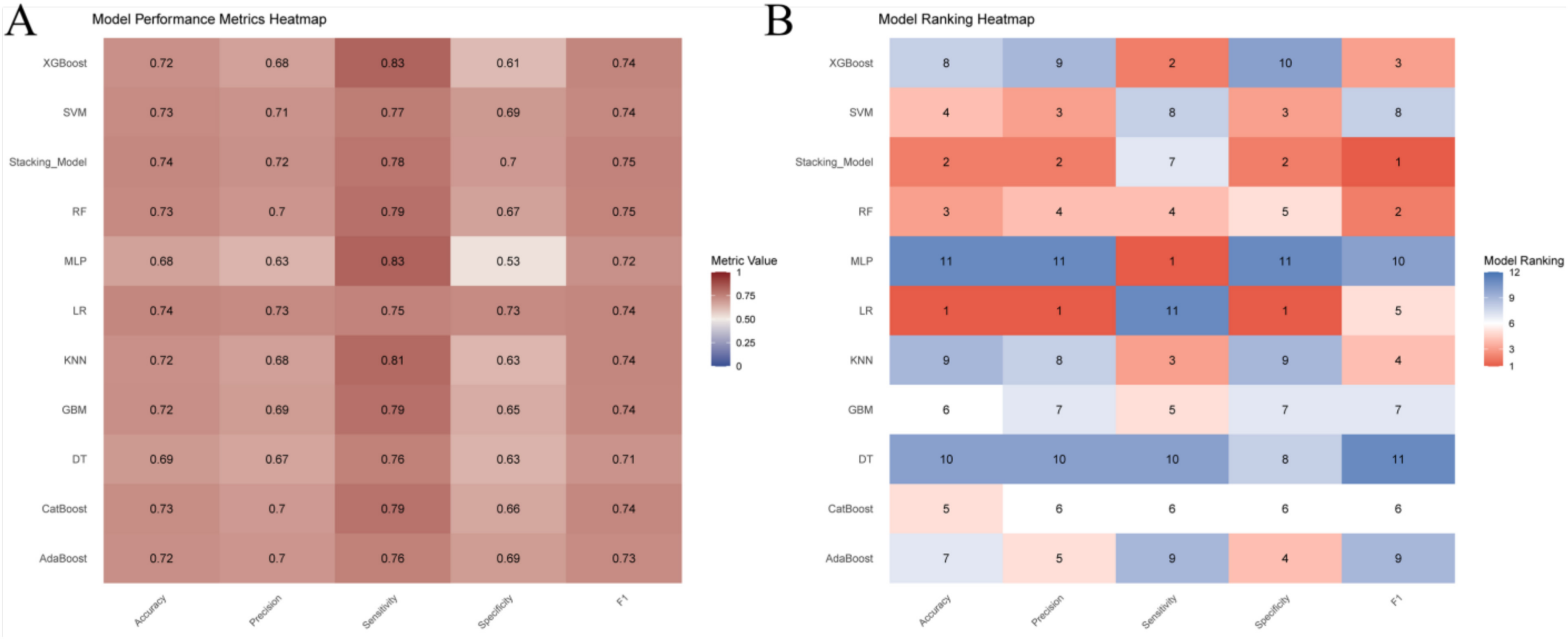
Performance evaluation of prediction models in the training cohort. **(A)** Comparison of model performance metrics, including accuracy, precision, sensitivity, specificity, and F1 score, across all prediction models in the training cohort; **(B)** ranking of the prediction models based on accuracy, precision, sensitivity, specificity, and F1 score in the training cohort.

Seventy percent of the dataset was randomly allocated to the training cohort. Based on the 14 selected predictors, ten machine learning algorithms were applied to develop individual base models for estimating the risk of ARDS in patients with intra-abdominal sepsis. These base learners were subsequently integrated into a stacked ensemble model using a stacking generalization framework. The stacking model demonstrated strong predictive performance, achieving an AUC of 0.811 (95% CI: 0.780–0.841). Calibration analysis showed close agreement between predicted and observed outcomes, with the calibration curve closely approximating the ideal reference line. DCA demonstrated that the stacking model provided a higher net benefit in the 80%–90% threshold range and indicated that the stacking model better identified high-risk patients, offering improved predictive accuracy and clinical utility, while the area under the PR curve reached 0.825 (95% CI: 0.785–0.859), reflecting robust discrimination and stability (Figure 3). In the training cohort, the stacking model achieved an overall accuracy of 0.740, with a precision of 0.717, sensitivity of 0.784, specificity of 0.697, and an F1 score of 0.749 (Figure 4), further supporting its balanced and reliable predictive capability.

### 3.4 Internal validation of prediction model performance

**FIGURE 5:**
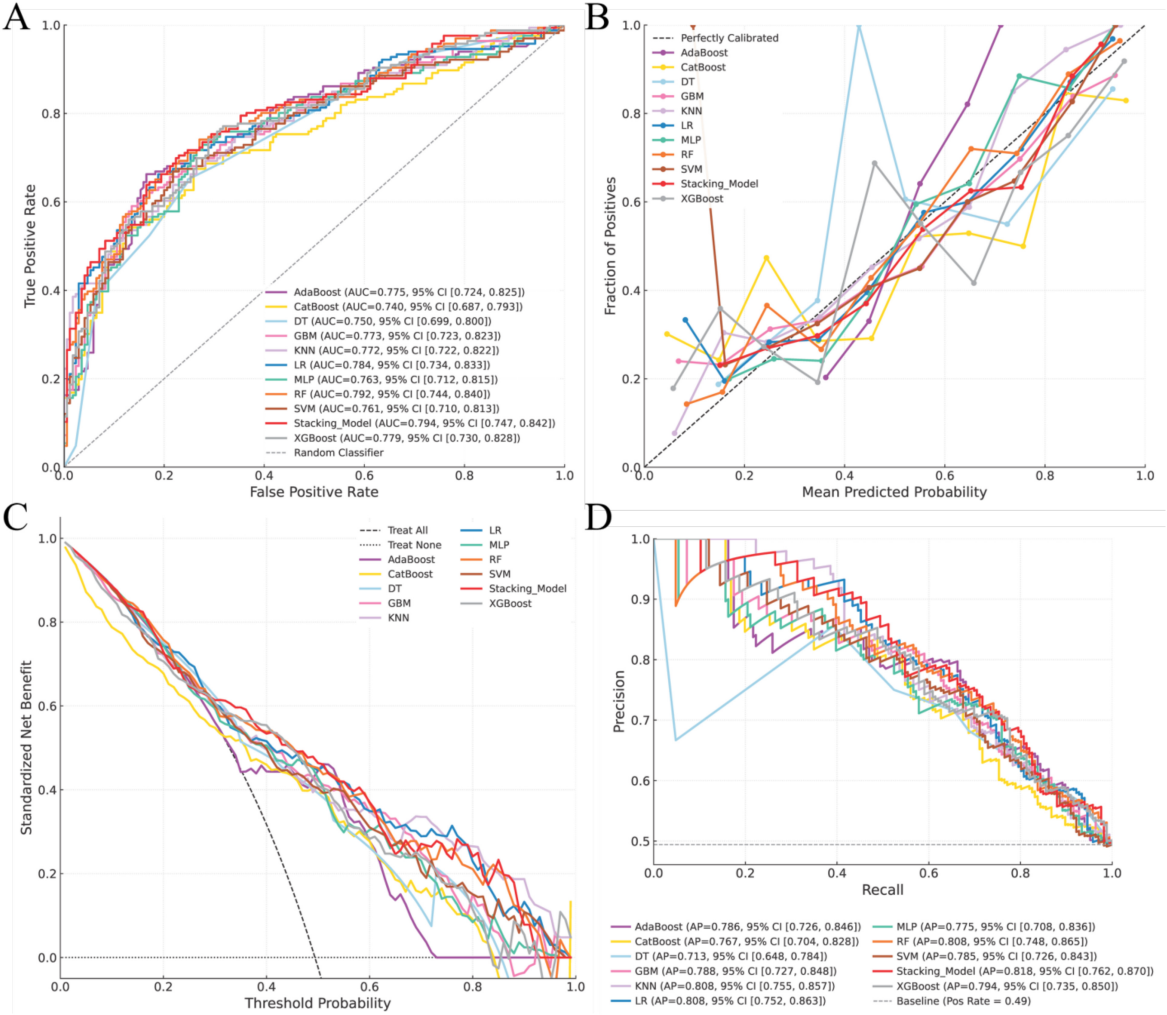
Performance of the prediction models in the internal validation cohort. **(A)** Receiver operating characteristic (ROC) curve for the internal validation cohort; **(B)** calibration curve assessing agreement between predicted and observed outcomes in the internal validation cohort; **(C)** decision curve analysis (DCA) evaluating the net clinical benefit across threshold probabilities in the internal validation cohort; **(D)** precision–recall (PR) curve for the internal validation cohort.

**FIGURE 6:**
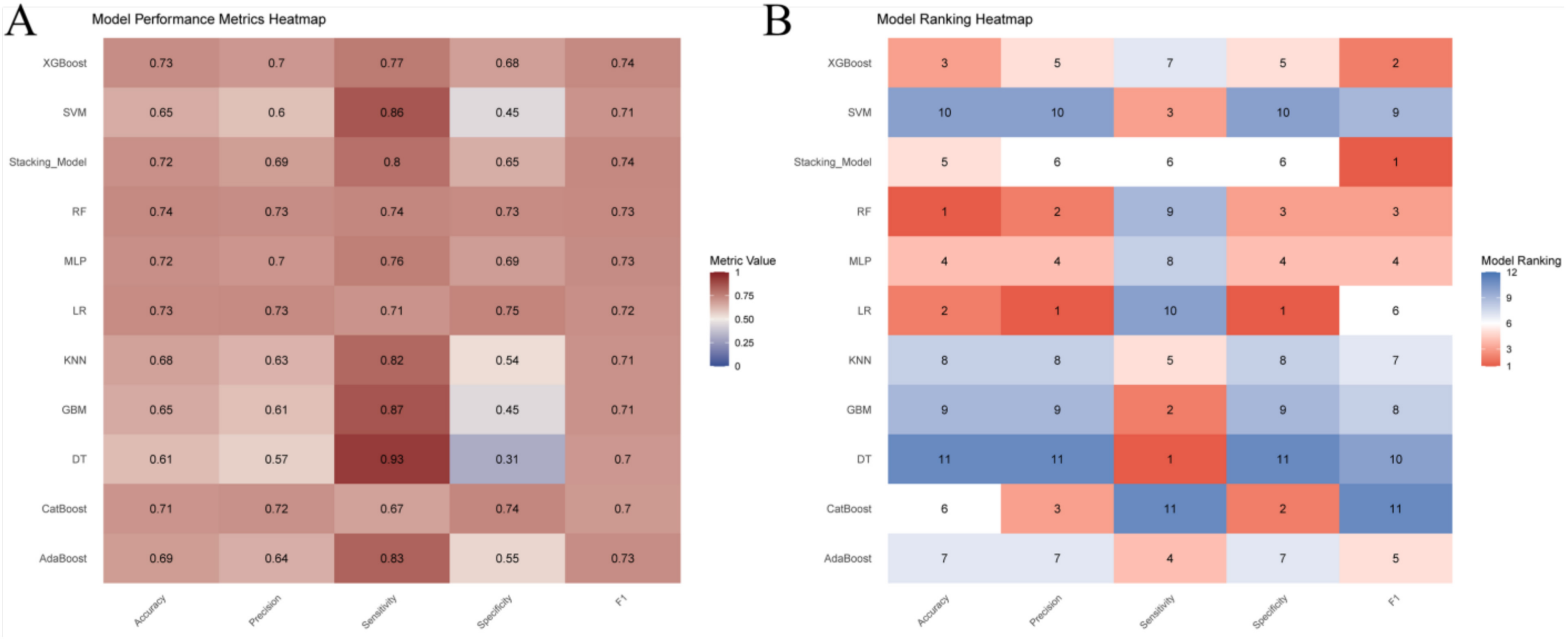
Performance evaluation of prediction models based on the internal validation cohort. **(A)** Comparison of model performance metrics, including accuracy, precision, sensitivity, specificity, and F1 score, across all prediction models in the internal validation cohort; **(B)** ranking of the prediction models based on accuracy, precision, sensitivity, specificity, and F1 score in the internal validation cohort.

The predictive performance of the model was internally assessed using a validation cohort comprising 30% of the original dataset, randomly selected. In this cohort, the stacking model achieved an AUC of 0.794 (95% CI: 0.747–0.842). Calibration analysis demonstrated good agreement between predicted probabilities and observed outcomes, with the calibration curve closely approximating the ideal reference line. DCA indicated a favorable net clinical benefit across a range of threshold probabilities. In addition, the area under the PR curve reached 0.818 (95% CI: 0.762–0.870), reflecting stable and reliable discriminative performance (Figure 5). With respect to classification metrics, the stacking model achieved an accuracy of 0.720, a precision of 0.688, a sensitivity of 0.795, a specificity of 0.647, and an F1 score of 0.737 in the internal validation cohort (Figure 6).

### 3.5 SHAP analysis for interpretation of the stacking model

**FIGURE 7:**
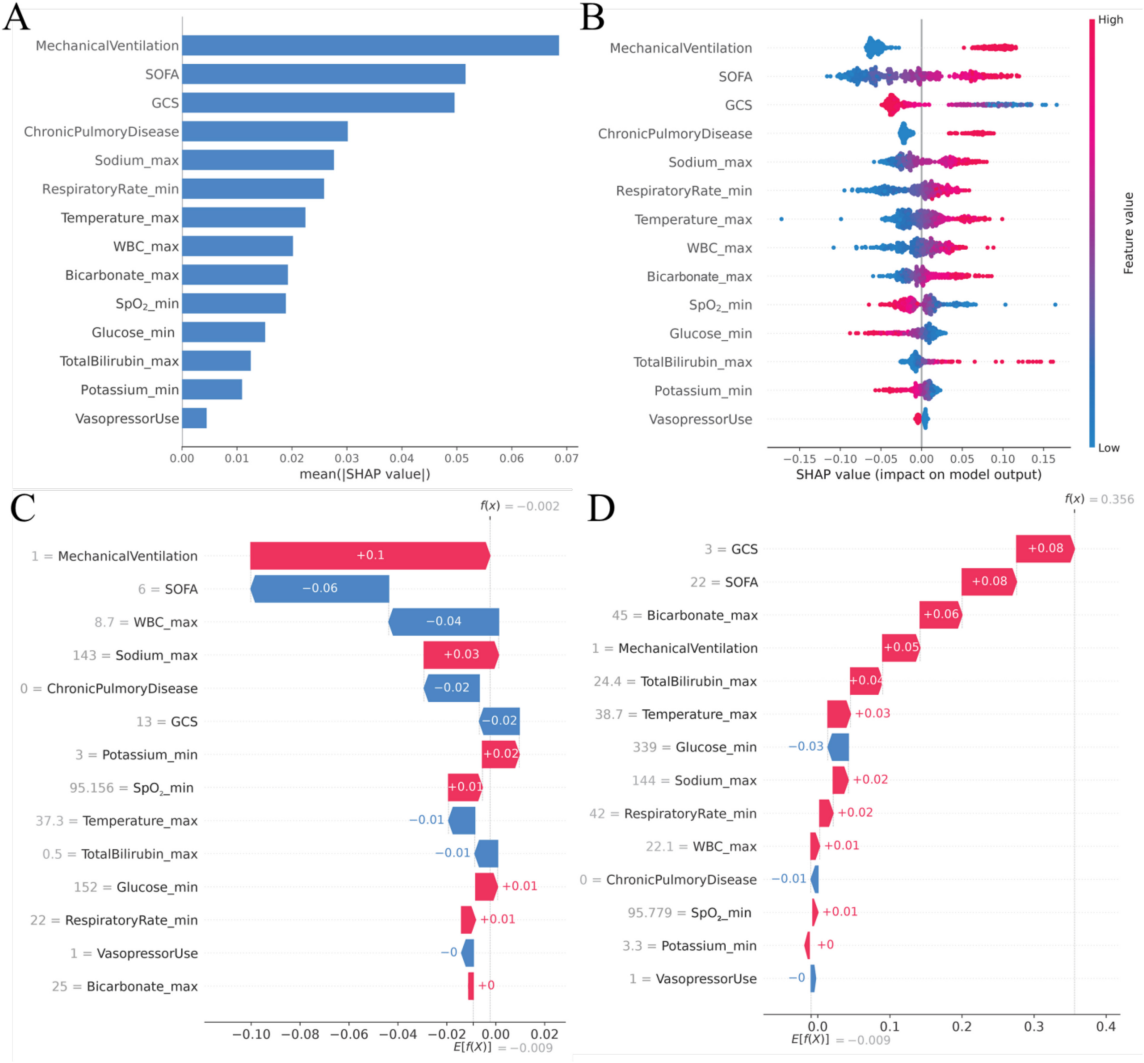
SHapley Additive exPlanations (SHAP) analysis of the stacking model. **(A)** Bar plot depicting the mean SHAP values of the 14 selected variables in the stacking model; **(B)** SHAP summary plot illustrating the distribution of SHAP values for the 14 key predictors; **(C)** waterfall plot demonstrating the individualized prediction and feature contributions for Patient Case 1; **(D)** waterfall plot demonstrating the individualized prediction and feature contributions for Patient Case 2.

**FIGURE 8:**
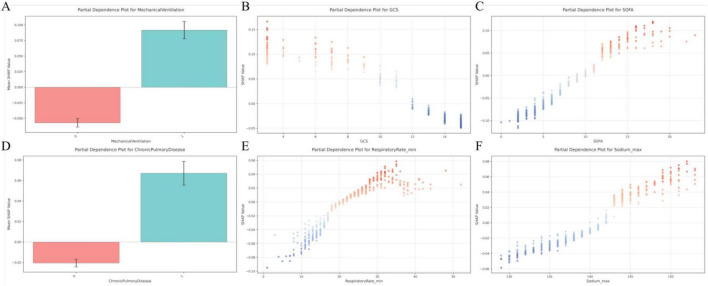
Partial dependence plots illustrating the effects of key variables on model predictions. **(A)** Relationship between mechanical ventilation status and mean SHapley Additive exPlanations (SHAP) values; **(B)** association between Glasgow Coma Scale (GCS) score and mean SHAP values; **(C)** association between Sequential Organ Failure Assessment (SOFA) score and mean SHAP values; **(D)** relationship between the presence of chronic pulmonary disease and mean SHAP values; **(E)** association between minimum respiratory rate and mean SHAP values; **(F)** association between maximum serum sodium concentration and mean SHAP values.

Figure 7A presents the mean SHAP values summarizing feature importance within the stacking model, revealing that mechanical ventilation had the strongest impact on predictions of ARDS. Other variables that contributed substantially to ARDS risk included higher SOFA score, lower GCS score, a history of chronic pulmonary disease, increased respiratory rate and body temperature, elevated white blood cell count and bicarbonate levels, reduced oxygen saturation, hypoglycemia, increased total bilirubin levels, hypokalemia, and hypernatremia. In contrast, early use of vasoactive agents was associated with a reduced predicted risk of ARDS (Figure 7B). To enhance interpretability at the individual level, waterfall plots were used to illustrate the cumulative and feature-specific contributions to model predictions, with representative patient cases provided as examples (Figures 7C, D). In addition, Figure 8 displays partial dependence plots for the six most influential predictors, further elucidating their effects on the model’s predictive output across varying levels of risk.

### 3.6 External validation of predictive model performance

**FIGURE 9:**
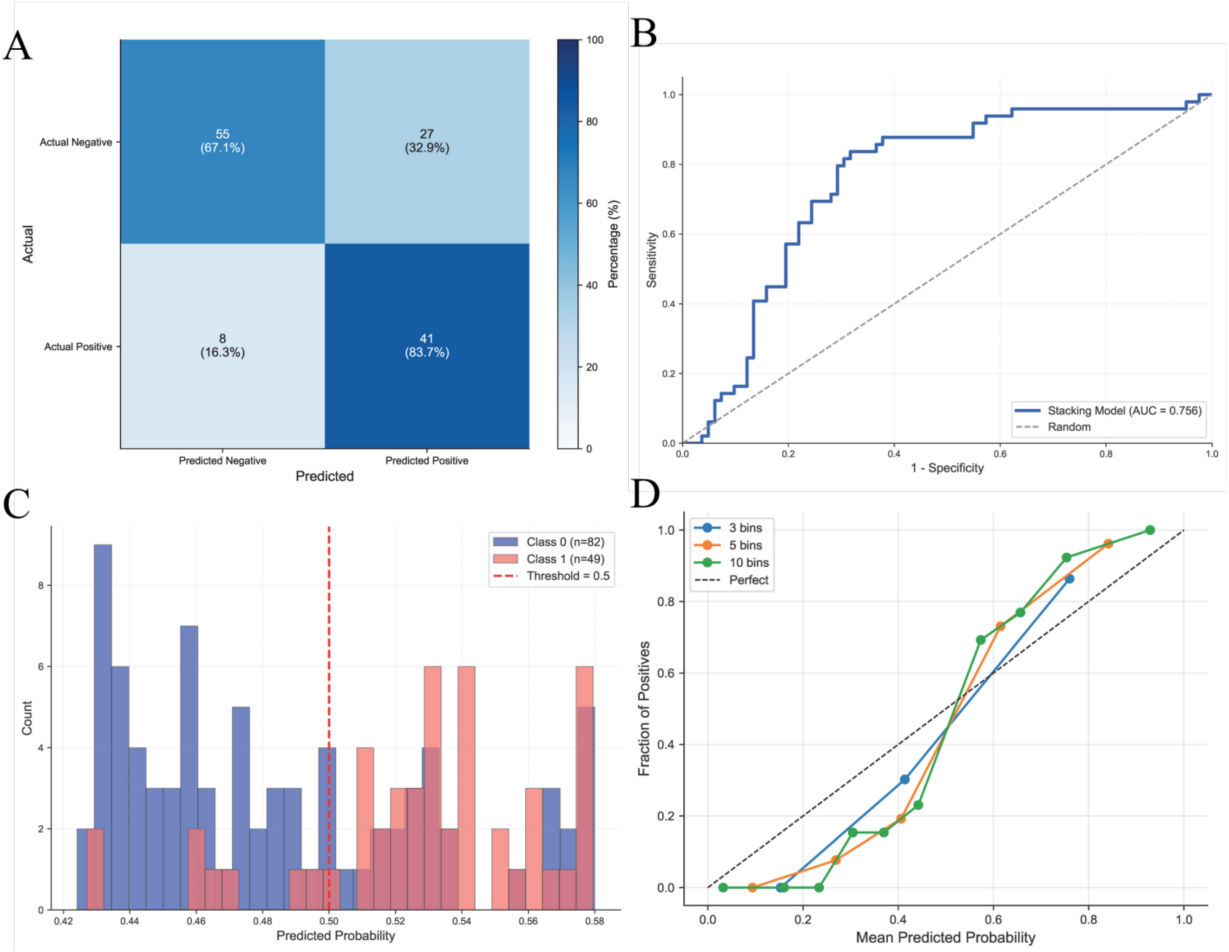
Performance of the predictive model in the external validation cohort. **(A)** Confusion matrix summarizing classification performance in the external validation cohort; **(B)** decision curve analysis (DCA) illustrating the net clinical benefit across threshold probabilities in the external validation cohort; **(C)** model discrimination in the external validation cohort, assessed by the Receiver operating characteristic (ROC) curve; **(D)** calibration curve demonstrating agreement between predicted probabilities and observed outcomes in the external validation cohort.

The predictive performance of the model was externally evaluated in an independent cohort of 131 patients with intra-abdominal sepsis admitted to the First Affiliated Hospital of Xinjiang Medical University (Supplementary Table 4). Confusion matrix based assessment showed that the stacking model achieved an overall classification accuracy of 73.28%, with an AUC of 0.756, indicating satisfactory discriminative performance. In addition, calibration curve analysis demonstrated close agreement between predicted risks and observed outcomes, supporting the stability and reliability of the model in the external validation cohort (Figure 9).

### 3.7 Development of a web-based prediction model calculator

To support real world clinical implementation, a web-based risk prediction calculator was developed based on the stacking model^3^. By entering routinely available clinical variables, users can obtain an individualized estimate of the probability of ARDS development in patients with intra-abdominal sepsis. The interface design and operational workflow of the online calculator are presented in Supplementary Figure 1.

## 4 Discussion

Intra-abdominal sepsis is a life-threatening infectious condition that typically originates from infections or injuries involving abdominal organs, such as peritonitis, gastrointestinal perforation, and intra-abdominal abscesses. Among its systemic complications, ARDS is one of the most common and severe, accounting for a substantial proportion of sepsis related morbidity and mortality (3, 7). In the present study, we initially constructed individual predictive models using 10 distinct machine learning algorithms. Building upon these base learners, an ensemble model was subsequently developed using a stacking strategy to provide a comprehensive risk prediction framework for ARDS in patients with intra-abdominal sepsis. The study population was restricted to patients admitted to the ICU with a confirmed diagnosis of intra-abdominal sepsis, thereby ensuring high clinical relevance to critically ill cohorts. Notably, the incidence of ARDS observed in this population was comparable to that reported in prior studies and was closely associated with adverse clinical outcomes, further underscoring the importance of early risk stratification and timely intervention in this vulnerable group (14). Ensemble learning approaches are specifically designed to integrate multiple base learners through weighted aggregation, allowing complementary strengths of individual algorithms to be leveraged while minimizing model-specific limitations. Such strategies can effectively reduce overfitting and improve predictive stability, robustness, and generalizability. Owing to these advantages, ensemble learning methods have been increasingly adopted in the development of clinical risk prediction models across diverse medical domains (12, 15). In this study, the proposed stacking model incorporated 14 routinely available and clinically accessible variables and demonstrated strong discriminative performance. Importantly, the reliance on standard clinical parameters ensures compatibility with real world clinical workflows, highlighting the model’s practicality and substantial potential for implementation as a clinical decision support tool.

Mechanical ventilation is a cornerstone of supportive care for patients with respiratory failure; however, it is also a well-recognized contributor to iatrogenic lung injury. Ventilator induced lung injury (VILI) results from a complex interaction between ventilator settings and patient-specific physiological characteristics, with its pathogenesis involving multiple mechanical and biological pathways (16). More recent investigations have expanded the mechanism framework by identifying additional contributors, such as pulmonary vascular injury, patient self-inflicted lung injury (P-SILI), and excessive mechanical power, which together provide a more comprehensive understanding of VILI development and progression (17, 18). In the present study, early exposure to mechanical ventilation was closely associated with the subsequent development of ARDS in patients with intra-abdominal sepsis, a finding that aligns with emerging evidence linking early ventilatory stress to lung injury in vulnerable populations (19). Patients with chronic pulmonary disease often exhibit reduced respiratory reserve and persistent airflow limitation. In this context, increased mucus secretion and exacerbated airway obstruction may precipitate dyspnea and wheezing. Such compromised pulmonary function not only predisposes these patients to ARDS but also emerged as a significant contributor to the predictive performance of our model (20). Furthermore, our analysis suggested that early use of vasoactive agents was associated with a reduced risk of ARDS, likely due to the benefits of prompt hemodynamic stabilization. Vasoactive therapy is essential in the management of septic shock, as it rapidly corrects hypotension, reduces tissue hypoperfusion, and minimizes the need for excessive fluid resuscitation. These effects may help prevent the complications associated with fluid overload, such as pulmonary edema, which could contribute to a reduced risk of ARDS. However, it is important to note that this finding reflects an association, not causality, and further studies are needed to confirm these potential benefits (21, 22).

The SOFA and GCS score are widely used to evaluate disease severity and prognosis in critically ill patients (23). In the context of sepsis, an elevated SOFA score in conjunction with a reduced GCS score reflects a vulnerable physiological state characterized by diminished organ reserve and impaired systemic resilience (24). The SOFA score offers a comprehensive evaluation of organ dysfunction, including respiratory, cardiovascular, renal, hepatic, coagulation, and neurological systems, and is routinely applied in the ICU for dynamic monitoring of disease progression and treatment response (7). Sepsis related systemic inflammation can disrupt the pulmonary capillary alveolar barrier, leading to increased vascular permeability, pulmonary edema, and ultimately the development of ARDS. A higher SOFA score therefore signifies more severe organ dysfunction, heightened inflammatory burden, and greater hemodynamic instability, all of which are closely associated with an increased risk of ARDS. Notably, the SOFA score incorporates the PaO_2_/FiO_2_ ratio, a core diagnostic criterion for ARDS, further reinforcing its relevance in early risk stratification (25). Consequently, an elevated SOFA score during the early phase of sepsis may serve as an important warning signal for impending ARDS. When sepsis triggers systemic inflammation and multiple organ dysfunction, impaired brain function often manifests as a decline in the GCS score. This reduction signifies not only greater illness severity but also a heightened inflammatory response, a central pathological mechanism in ARDS (26). Patients with a low GCS score also frequently exhibit impaired gag and cough reflexes, increasing the risk of aspiration, pulmonary infection, and inhalation injury, all significant risk factors for ARDS (26). The SHAP analysis in this study confirms that both the SOFA and GCS score are critical variables that substantially improve the model’s predictive performance.

SHapley Additive exPlanations analysis highlighted elevated respiratory rate, decreased oxygen saturation, and increased body temperature as strong predictors of ARDS in patients with intra-abdominal sepsis. These vital signs are routinely and continuously monitored in the ICU, and their temporal fluctuations provide direct insight into a patient’s evolving physiological status, thereby offering clinicians timely and actionable information to support individualized management strategies (27). Consistent with our findings, previous studies have identified abnormalities in these parameters as key indicators of disease severity and adverse outcomes in ARDS, underscoring their established clinical and prognostic relevance (28, 29). Although vital signs are susceptible to non-specific influences and require interpretation within the appropriate clinical context, their universal availability and real-time nature confer substantial practical advantages. When incorporated into machine learning based prediction models, continuous vital sign data offer a simple, cost-effective, and sensitive approach to early risk stratification. This integration substantially enhances the feasibility of implementing predictive tools in routine clinical practice and supports timely identification of patients at high risk for ARDS (30).

In the development of this predictive model, routinely obtained laboratory parameters provided critical insight into patients’ physiological status and underlying pathophysiology. SHAP analysis identified several laboratory variables with substantial predictive contributions, including white blood cell count, bicarbonate, blood glucose, serum potassium, electrolyte disturbances, and elevated total bilirubin levels. Collectively, these indicators reflect key mechanisms such as infectious and inflammatory responses, metabolic derangements, organ dysfunction, and acid base imbalance, all of which are closely linked to the development and prognosis of ARDS (22). White blood cell count demonstrated a strong early association with mortality risk, suggesting that extreme values may signify either an overwhelming inflammatory response or a state of sepsis associated immunosuppression (31). Bicarbonate, a central component of the body’s primary buffering system, is highly sensitive to disturbances in acid–base homeostasis, and both elevated and reduced levels have been associated with impaired respiratory function and adverse outcomes (32). Hepatic dysfunction, which compromises the clearance of inflammatory mediators, coagulation factor synthesis, and immune regulation, can further exacerbate pulmonary inflammation and injury (33). Accordingly, elevated total bilirubin serves as an important indicator of organ damage in septic patients. Prior studies have shown that in sepsis, particularly in the presence of mechanical ventilation or tissue hypoperfusion, hepatocellular injury is often intensified, leading to impaired bilirubin excretion and serum levels that correlate strongly with increased mortality risk (34). Although the direct relationship between isolated hypoglycemia and ARDS remains insufficiently characterized, hypoglycemia has been widely recognized as a marker of poor prognosis and is incorporated into several critical illness scoring systems (35). Electrolyte abnormalities, including hypokalemia and hypernatremia, can disrupt cellular electrophysiological stability and impair respiratory muscle function, thereby increasing susceptibility to respiratory failure (36). Moreover, such electrolyte disturbances may reflect complex underlying pathophysiological processes, including inflammation mediated shifts in extracellular fluid distribution and dysfunction of the adrenal and renal systems, further supporting their relevance as predictive variables (37). In multivariable predictive models, integrating these laboratory indicators with established disease severity scores enables a more comprehensive and quantitative assessment of organ function and metabolic status. This integrative approach enhances early risk stratification and improves the timely identification of patients at increased risk for ARDS.

In summary, the prediction model developed using an ensemble learning framework, integrating routinely applied ICU severity scores and readily available clinical indicators, demonstrated strong discriminative performance and substantial potential for clinical interpretability and real world application. By leveraging early ICU data, the model provides a practical tool for timely risk stratification in patients with intra-abdominal sepsis. Nevertheless, several limitations warrant consideration. First, the model was constructed using clinical variables collected on the first day of ICU admission, with the intent of enabling early identification of patients at high risk based on readily accessible monitoring data. In routine clinical practice, however, incorporating longitudinal trends and dynamic changes in patient parameters may yield a more comprehensive and accurate assessment. Second, although the model was developed and validated using large, multicenter datasets from the MIMIC-IV and eICU-CRD, the external cohort was a single-center study with potential differences in case mix, care patterns, and its generalizability remains to be confirmed through external prospective validation in diverse clinical settings. Third, the stacking ensemble employed logistic regression as the meta-learner; future investigations could explore the incorporation of more advanced modeling techniques to further enhance predictive performance. Fourth, due to the potential temporal and geographic drift, the practices, including sepsis management, have evolved from 2016 to 2024, which may affect the model’s performance over time or across regions. The model’s ability to maintain its predictive accuracy in the face of changing clinical practices and regional differences remains an important area for future validation and refinement. Finally, the present study did not stratify patients according to ARDS severity. Given the substantial clinical heterogeneity among septic patients, future research should examine differences in treatment response and outcomes across varying degrees of ARDS severity.

## 5 Conclusion

This study developed and externally validated an ensemble learning based prediction model to estimate the risk of ARDS in patients with intra-abdominal sepsis, integrating routinely used clinical indicators and severity scores. The model demonstrated robust discriminative performance and calibration stability, providing clinically meaningful insights for early risk stratification and therapeutic decision-making. By enabling timely identification of patients at elevated risk for ARDS, the proposed model has the potential to support individualized clinical management, improve resource utilization, and ultimately enhance patient outcomes.

## Data Availability

The raw data supporting the conclusions of this article will be made available by the authors, without undue reservation.

## Footnotes

1
https://www.postgresql.org/download/

2
https://www.navicat.com/en/download/navicat-premium

3
https://intra-abdominal-sepsis-ards-risk-predictor-001.streamlit.app/
