# Supplementary material for: Development and validation of a prognostic model for acute respiratory distress syndrome in critically Ill patients with intra-abdominal sepsis: a multicenter cohort study
Source: Front Med (Lausanne). 2026 Mar 12;13:1775636. doi: 10.3389/fmed.2026.1775636 (PMC13017791; doi:10.3389/fmed.2026.1775636)
Supplement: Supplementary file 1 [file Image_1.pdf]

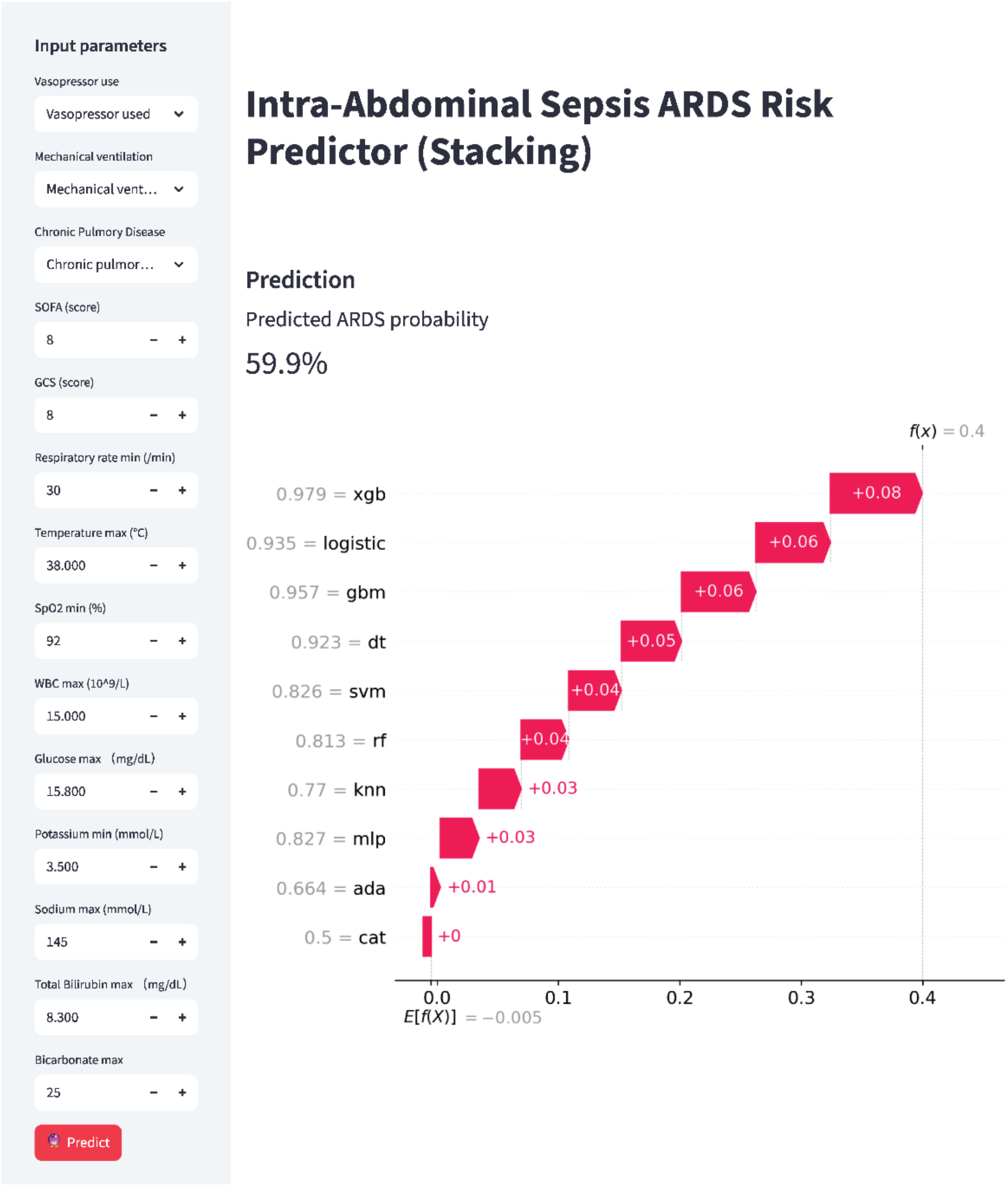

Supplementary Figure 1. A Web-based prediction tool for estimating the risk of ARDS in patients with intra-abdominal sepsis.
