# Supplementary material for: Development and validation of a prognostic model for acute respiratory distress syndrome in critically Ill patients with intra-abdominal sepsis: a multicenter cohort study
Source: Front Med (Lausanne). 2026 Mar 12;13:1775636. doi: 10.3389/fmed.2026.1775636 (PMC13017791; doi:10.3389/fmed.2026.1775636)
Supplement: Supplementary file 2 [file Table_1.docx]

**Supplementary Table 1.** Identification of Intra-Abdominal Infection Across Cohorts

| **Cohorts** | **ICD-9/ICD-10 Codes** |
| --- | --- |
| **MIMIC-IV Cohort** | **ICD-9:** icd_code = '5400' OR icd_code = '5409' OR icd_code = '5671' OR icd_code = '56723' OR icd_code = '56729' OR icd_code = '56789' OR icd_code = '800' OR icd_code = '801' OR icd_code = '802' OR icd_code = '803' OR icd_code = '804' OR icd_code = '809' OR icd_code = '81' OR icd_code = '82' OR icd_code = '83' OR icd_code = '841' OR icd_code = '842' OR icd_code = '843' OR icd_code = '844' OR icd_code = '845' OR icd_code = '846' OR icd_code = '847' OR icd_code = '849' OR icd_code = '88' )  **ICD-10:** icd_code = 'K352' OR icd_code = 'K3520' OR icd_code = 'K3521' OR icd_code = 'K353' OR icd_code = 'K3530' OR icd_code = 'K3531' OR icd_code = 'K3532' OR icd_code = 'K3533' OR icd_code = 'K650' OR icd_code = 'K652' OR icd_code = 'K658' OR icd_code = 'A04' |
| **eICU-CRD Cohort** | **ICD-9/ICD-10**: icd_code = ‘008.45, A04.7’ OR icd_code = ‘567.22, K65.1’ OR icd_code = ‘567.9, K65.0’ OR icd_code = ‘562.11, K57.32’ OR icd_code = ‘573.1, 078.5, B25.1’ OR icd_code = ‘567.21, K65.2’ OR icd_code = ‘575.0, K81.0’ OR icd_code = ‘567.21, K65.0’ OR icd_code = ‘530.10, K20.9’ OR icd_code = ‘K61.1’ OR icd_code = ‘998.59’ OR icd_code = ‘574.00, K81.0’ OR icd_code = ‘567.23, K65.2’ OR icd_code = ‘009.1, A09’ OR icd_code = ‘576.1, K83.0’ OR icd_code = ‘541, K35.80’ OR icd_code = ‘577.0, K85.8’ OR icd_code = ‘572.0, K75.0’ OR icd_code = ‘569.5, K63.0’ OR icd_code = ‘070.51, 573.1, B19.10’ OR icd_code = ‘573.1, 070.30, B17.1’ OR icd_code = ‘569.5’ OR icd_code = ‘540.1, K35.3’ OR icd_code = ‘567.38, K68.1’ OR icd_code = ‘075, 573.1, B27.09’ OR icd_code = ‘540.0, K35.2’ OR icd_code = ‘577.0, K65.1’ OR icd_code = ‘573.1, B17.9’ OR icd_code = ‘289.59, D73.3’ OR icd_code = ‘575.11, K81.0’ OR icd_code = ‘567.9’ OR icd_code = ‘573.1, 070.1, B15.9’ OR icd_code = ‘004.9, A03.9’ OR icd_code = ‘005.9, A05.9’ OR icd_code = ‘567.21, T81.6’ OR icd_code = ‘567.22, D73.3’ OR icd_code = ‘567.38, K68.19’ |
| **XJMU Cohort** | **ICD-9:** icd_code = '5400' OR icd_code = '5409' OR icd_code = '5671' OR icd_code = '56723' OR icd_code = '56729' OR icd_code = '56789' OR icd_code = '800' OR icd_code = '801' OR icd_code = '802' OR icd_code = '803' OR icd_code = '804' OR icd_code = '809' OR icd_code = '81' OR icd_code = '82' OR icd_code = '83' OR icd_code = '841' OR icd_code = '842' OR icd_code = '843' OR icd_code = '844' OR icd_code = '845' OR icd_code = '846' OR icd_code = '847' OR icd_code = '849' OR icd_code = '88' )  **ICD-10:** icd_code = 'K352' OR icd_code = 'K3520' OR icd_code = 'K3521' OR icd_code = 'K353' OR icd_code = 'K3530' OR icd_code = 'K3531' OR icd_code = 'K3532' OR icd_code = 'K3533' OR icd_code = 'K650' OR icd_code = 'K652' OR icd_code = 'K658' OR icd_code = 'A04' |

The identification of intra-abdominal infections across the cohorts was standardized by mapping ICD-9 and ICD-10 codes for relevant diagnoses (e.g., appendicitis, peritonitis, and other abdominal pathologies). Specific codes used to identify infections in the MIMIC-IV, eICU-CRD, and XJMU cohorts are listed, with coding strategies adjusted for each dataset's structure and diagnostic practices. This approach ensures that the diagnosis of intra-abdominal infection is consistent across different datasets despite variations in clinical documentation and coding practices.
