# Supplementary material for: Development and validation of a prognostic model for acute respiratory distress syndrome in critically Ill patients with intra-abdominal sepsis: a multicenter cohort study
Source: Front Med (Lausanne). 2026 Mar 12;13:1775636. doi: 10.3389/fmed.2026.1775636 (PMC13017791; doi:10.3389/fmed.2026.1775636)
Supplement: Supplementary file 3 [file Table_2.docx]

**Supplementary Table 2.** Operationalization of the Berlin Definition for ARDS Across Cohorts

| **Berlin Definition Component** | **Operational Criterion** | **MIMIC-IV Cohort** | **eICU-CRD Cohort** | **XJMU Cohort** | **Missing Data Handling** |
| --- | --- | --- | --- | --- | --- |
| **Timing** | ICU admission time + 24h | ICU admission time + 24h | ICU admission time + 24h | ICU admission time + 24h | Not applicable |
| **Oxygenation** | PaO₂/FiO₂ ≤ 300 mmHg | PaO₂ from labevents; FiO₂ from respiratory chart; lowest ratio during ICU stay | PaO₂ from labevents; FiO₂ from respiratory chart; lowest ratio during ICU stay | ABG record in EMR | If FiO₂ missing, case not considered ARDS unless complete ABG available |
| **PEEP Requirement** | PEEP ≥ 5 cmH₂O | Extracted from ventilator settings or HFNC flow > 30 L/min | Extracted from ventilator settings or HFNC flow > 30 L/min | Ventilator or oxygen therapy record | Cases without documented PEEP excluded |
| **Radiographic Findings** | Bilateral infiltrates not fully explained by effusion/collapse | The free-text radiology reports | The diagnosis code of radiology report | Imaging report documentation | Text-based search strategy applied. If imaging unavailable, ARDS not confirmed |
| **Exclusion of Hydrostatic Edema** | No primary cardiogenic pulmonary edema | Excluded if primary HF diagnosis or cardiogenic shock | Excluded if primary HF diagnosis or cardiogenic shock | Excluded if primary HF diagnosis or cardiogenic shock | Not applicable |

The Berlin Definition of ARDS was operationalized consistently across all three cohorts (MIMIC-IV, eICU-CRD, and XJMU cohorts) using key components such as oxygenation levels (PaO₂/FiO₂ ≤ 300 mmHg), PEEP requirements (≥5 cmH₂O), radiographic findings (bilateral infiltrates), and exclusion of hydrostatic edema. These criteria were adapted according to the available clinical data, including ventilator settings, ABG records, and radiology reports. Missing data handling was applied by excluding cases without complete information on oxygenation or PEEP. Arterial blood gas analysis (ABG), Electronic medical record (EMR), high flow nasal cannula therapy (HFNC), heart failure (HF)
