# Supplementary material for: Development and validation of a prognostic model for acute respiratory distress syndrome in critically Ill patients with intra-abdominal sepsis: a multicenter cohort study
Source: Front Med (Lausanne). 2026 Mar 12;13:1775636. doi: 10.3389/fmed.2026.1775636 (PMC13017791; doi:10.3389/fmed.2026.1775636)
Supplement: Supplementary file 4 [file Table_3.docx]

**Supplementary Table 3.** Hyperparameters of each Machine Learning model.

| **Models** | **Hyperparameters** |
| --- | --- |
| Adaptive Boosting | clf__algorithm="SAMME.R"; clf__learning_rate=0.1; clf__n_estimators=400; clf__base_estimator=DecisionTreeClassifier(max_depth=1); clf__estimator__max_depth=1; clf__estimator__criterion="gini"; clf__estimator__splitter="best"; clf__estimator__min_samples_split=2; clf__estimator__min_samples_leaf=1; clf__random_state=42 |
| Categorical Boosting | clf__depth=6; clf__iterations=1200; clf__learning_rate=0.05; clf__l2_leaf_reg=3; clf__bootstrap_type=Bayesian; clf__loss_function=Logloss; clf__eval_metric=AUC; clf__random_strength=1.0; clf__bagging_temperature=1.0; clf__border_count=254; clf__grow_policy=SymmetricTree; clf__min_data_in_leaf=1; clf__random_state=42; clf__verbose=False |
| Decision Tree | clf__criterion="gini"; clf__splitter="best"; clf__max_depth=3; clf__min_samples_split=2; clf__min_samples_leaf=1; clf__min_weight_fraction_leaf=0.0; clf__max_features=None; clf__max_leaf_nodes=None; clf__min_impurity_decrease=0.0; clf__class_weight=None; clf__random_state=42 |
| Gradient Boosting Machine | clf__learning_rate=0.1; clf__n_estimators=300; clf__max_depth=3; clf__criterion="friedman_mse"; clf__min_samples_split=2; clf__min_samples_leaf=1; clf__min_weight_fraction_leaf=0.0; clf__subsample=1.0; clf__max_features=None; clf__max_leaf_nodes=None; clf__min_impurity_decrease=0.0; clf__random_state=42 |
| k-Nearest Neighbors | clf__n_neighbors=5; clf__p=1; clf__weights="distance"; clf__algorithm="auto"; clf__leaf_size=30; clf__metric="minkowski"; clf__metric_params=None; clf__n_jobs=-1 |
| Logistic Regression | clf__C=1; clf__penalty="l1"; clf__class_weight="balanced"; clf__solver="liblinear"; clf__fit_intercept=True; clf__intercept_scaling=1; clf__max_iter=1000; clf__tol=0.0001; clf__warm_start=False; clf__random_state=42 |
| Multilayer Perceptron | clf__hidden_layer_sizes=(64,32); clf__activation="relu"; clf__solver="adam"; clf__alpha=0.0001; clf__learning_rate="constant"; clf__learning_rate_init=0.001; clf__batch_size="auto"; clf__max_iter=500; clf__shuffle=True; clf__tol=0.0001; clf__early_stopping=False; clf__beta_1=0.9; clf__beta_2=0.999; clf__epsilon=1e-08; clf__random_state=42 |
| Random Forest | clf__n_estimators=600; clf__max_depth=None; clf__min_samples_split=2; clf__min_samples_leaf=1; clf__min_weight_fraction_leaf=0.0; clf__max_features="auto"; clf__max_leaf_nodes=None; clf__bootstrap=True; clf__criterion="gini"; clf__class_weight=None; clf__random_state=42; clf__n_jobs=-1 |
| Support Vector Machine | clf__C=10; clf__kernel="rbf"; clf__gamma="scale"; clf__class_weight=None; clf__degree=3; clf__coef0=0.0; clf__shrinking=True; clf__probability=True; clf__tol=0.001; clf__cache_size=200; clf__max_iter=-1; clf__random_state=42 |
| Extreme Gradient Boosting | clf__learning_rate=0.05; clf__max_depth=3; clf__subsample=0.7; clf__colsample_bytree=0.7; clf__reg_lambda=1; clf__n_estimators=400; clf__gamma=0; clf__min_child_weight=1; clf__objective="binary:logistic"; clf__eval_metric="auc"; clf__tree_method="auto"; clf__random_state=42 |
| stacked ensemble model | clf__penalty="l2"; clf__solver="lbfgs"; clf__C=0.428; clf__max_iter=5000; clf__fit_intercept=True; clf__intercept_scaling=1.0; clf__class_weight=None; clf__tol=0.0001; clf__warm_start=False; clf__n_jobs=-1; clf__random_state=42 |
