# Supplementary material for: Development and validation of a prognostic model for acute respiratory distress syndrome in critically Ill patients with intra-abdominal sepsis: a multicenter cohort study
Source: Front Med (Lausanne). 2026 Mar 12;13:1775636. doi: 10.3389/fmed.2026.1775636 (PMC13017791; doi:10.3389/fmed.2026.1775636)
Supplement: Supplementary file 5 [file Table_4.docx]

**Supplementary Table 4.** Characteristics of the Study Population in the First Affiliated Hospital of Xinjiang Medical University Cohort.

| **Variables** | **All Patients (n=131)** | **Non-ARDS Patients (n=82)** | | **ARDS Patients (n=49)** | ***p* Value** |
| --- | --- | --- | --- | --- | --- |
| Age (years) | 62.6 ± 11.2 | | 62.9 ± 10.9 | 62.1 ± 11.7 | 0.687 |
| Gender (Male) | 56 (42.7%) | | 39 (47.6%) | 17 (34.7%) | 0.208 |
| Height (cm) | 168 ± 8.85 | | 169 ± 7.93 | 166 ± 10.1 | 0.13 |
| Charlson Comorbidity Index | 4.10 ± 2.37 | | 3.48 ± 2.26 | 5.13 ± 2.20 | <0.001 |
| SOFA Score | 6.51 ± 3.54 | | 5.01 ± 2.48 | 9.03 ± 3.63 | <0.001 |
| APACHE II Score | 20.8 ± 9.22 | | 16.1 ± 5.89 | 28.5 ± 8.59 | <0.001 |
| GCS Score | 10.1 ± 4.04 | | 12.3 ± 3.17 | 6.36 ± 2.15 | <0.001 |
| Comorbidities |  | |  |  |  |
| Congestive Heart Failure | 15 (11.5%) | | 8 (9.8%) | 7 (14.3%) | 0.614 |
| Chronic Pulmonary Disease | 1 (0.8%) | | 0 (0%) | 1 (2.0%) | 0.794 |
| Diabetes | 53 (40.5%) | | 22 (26.8%) | 31 (63.3%) | <0.001 |
| Chronic Kidney Disease | 28 (21.4%) | | 3 (3.7%) | 25 (51.0%) | <0.001 |
| Metastatic solid tumor | 10 (7.6%) | | 6 (7.3%) | 4 (8.2%) | 1 |
| Vital Signs |  | |  |  |  |
| Minimum Heart Rate (beats/min) | 63.0 [43.9, 83.7] | | 66.2 [43.9, 83.7] | 59.8 [45.9, 78.2] | 0.002 |
| Maximum Heart Rate (beats/min) | 122 [89.4, 178] | | 118 [89.4, 178] | 131 [95.2, 174] | 0.001 |
| Minimum Systolic Blood Pressure (mmHg) | 76.5 [29.0, 115] | | 78.2 [29.0, 112] | 73.2 [33.4, 115] | 0.007 |
| Maximum Systolic Blood Pressure (mmHg) | 163 [117, 218] | | 158 [117, 218] | 169 [131, 209] | 0.006 |
| Minimum Diastolic Blood Pressure (mmHg) | 41.4 [21.6, 66.5] | | 42.3 [22.0, 66.5] | 40.0 [21.6, 53.5] | <0.001 |
| Maximum Diastolic Blood Pressure (mmHg) | 92.8 [68.1, 136] | | 92.0 [68.1, 136] | 95.1 [78.9, 118] | 0.184 |
| Minimum Respiratory Rate (breaths/min) | 9.11 [0, 17.9] | | 9.90 [0.0627, 15.7] | 6.30 [0, 17.9] | <0.001 |
| Maximum Respiratory Rate (breaths/min) | 38.8 [21.5, 58.0] | | 37.3 [21.5, 54.3] | 43.3 [29.7, 58.0] | <0.001 |
| Minimum Temperature (°C) | 35.7 [27.6, 36.6] | | 36.1 [29.2, 36.6] | 33.4 [27.6, 36.3] | <0.001 |
| Maximum Temperature (°C) | 38.0 [37.0, 40.4] | | 37.8 [37.2, 39.8] | 38.5 [37.0, 40.4] | <0.001 |
| Minimum SpO₂ (%) | 86.3 [16.9, 93.8] | | 88.3 [52.2, 93.8] | 79.3 [16.9, 91.3] | <0.001 |
| Laboratory Parameters |  | |  |  |  |
| Maximum White Blood Cell Count (10⁹/L) | 19.4 [10.5, 59.2] | | 17.6 [10.5, 44.9] | 22.1 [13.4, 59.2] | 0.04 |
| Minimum Hemoglobin (g/dL) | 8.17 [5.03, 11.4] | | 8.49 [6.31, 11.4] | 7.62 [5.03, 11.4] | 0.006 |
| Minimum Hematocrit (%) | 25.4 [15.9, 35.2] | | 26.3 [19.1, 33.7] | 23.8 [15.9, 35.2] | 0.016 |
| Minimum Platelet Count (10⁹/L) | 179 [11.9, 474] | | 205 [27.0, 474] | 138 [11.9, 359] | 0.001 |
| Maximum Blood Urea Nitrogen (mg/dL) | 35.0 [2.49, 113] | | 29.8 [2.49, 113] | 50.1 [13.7, 108] | <0.001 |
| Maximum Serum Creatinine (mg/dL) | 1.96 [0.871, 8.51] | | 1.68 [0.871, 6.77] | 2.39 [1.01, 8.51] | 0.124 |
| Minimum Serum Albumin (g/dL) | 1.92 [1.16, 2.59] | | 1.96 [1.16, 2.51] | 1.88 [1.27, 2.59] | 0.462 |
| Maximum Sodium (mmol/L) | 143 [137, 152] | | 142 [137, 148] | 146 [141, 152] | <0.001 |
| Minimum Potassium (mmol/L) | 3.27 [2.72, 3.97] | | 3.31 [2.72, 3.97] | 3.19 [2.75, 3.89] | 0.029 |
| Maximum Potassium (mmol/L) | 4.60 [4.01, 5.80] | | 4.51 [4.01, 5.80] | 4.83 [4.27, 5.80] | <0.001 |
| Minimum HCO₃⁻ | 19.5 [6.00, 27.0] | | 20.1 [13.0, 27.0] | 17.9 [6.00, 25.8] | <0.001 |
| Maximum HCO₃⁻ | 30.1 [21.4, 37.6] | | 28.8 [21.4, 33.4] | 31.5 [27.0, 37.6] | <0.001 |
| Minimum Blood Glucose (mg/dL) | 80.8 [42.8, 146] | | 87.2 [42.8, 146] | 66.7 [47.6, 122] | 0.005 |
| Maximum Blood Glucose (mg/dL) | 234 [112, 380] | | 224 [112, 380] | 251 [134, 341] | 0.143 |
| Maximum Alanine Aminotransferase (U/L) | 43.1 [15.1, 222] | | 39.3 [15.1, 147] | 61.1 [15.1, 222] | <0.001 |
| Maximum Aspartate Aminotransferase (U/L) | 60.4 [18.1, 397] | | 44.9 [18.1, 260] | 112 [20.1, 397] | <0.001 |
| Maximum Total Bilirubin (mg/dl) | 0.925 [0.305, 13.0] | | 0.931 [0.428, 11.2] | 0.862 [0.305, 13.0] | 0.028 |
| Maximum INR | 1.50 [1.00, 3.28] | | 1.44 [1.10, 3.28] | 1.65 [1.00, 3.18] | 0.029 |
| Maximum APTT (s) | 35.6 [26.4, 98.1] | | 33.7 [27.2, 52.8] | 44.8 [26.4, 98.1] | <0.001 |
| Use of Vasopressors | 73 (55.7%) | | 43 (52.4%) | 30 (61.2%) | 0.425 |
| Use of Mechanical Ventilation | 52 (39.7%) | | 34 (41.5%) | 18 (36.7%) | 0.726 |
| Total Length of Stay (days) | 21.3 (28.6) | | 12.8 (9.41) | 35.4 (41.7) | <0.001 |
| ICU Length of Stay (days) | 10.8 (22.0) | | 4.19 (3.06) | 21.9 (33.1) | <0.001 |
| 28-day Mortality | 13 (9.9%) | | 4 (4.9%) | 9 (18.4%) | 0.028 |
| In-hospital Mortality | 20 (15.3%) | 5 (6.1%) | | 15 (30.6%) | <0.001 |
| ICU Mortality | 4 (3.1%) | 4 (4.9%) | | 0 (0%) | 0.296 |

Data shown as mean ± SD, median [min, max], or numbers with %.
